# Fracture incidence in women with polycystic ovarian syndrome: A retrospective cohort analysis

**DOI:** 10.1016/j.jcot.2026.103433

**Published:** 2026-04-09

**Authors:** Tanya Li, Wali U. Pirzada, Ameera Syed, Simran Shamith, Kimberly Dong, Asif M. Ilyas, Meghan Bishop

**Affiliations:** a Drexel University College of Medicine, 60 N 36th St, Philadelphia, PA, 19104, USA; b Rothman Orthopaedic Institute, 33 S. 9th St, Suite 1220, Philadelphia, PA, 19107, USA; c Rowan-Virtua School of Osteopathic Medicine, 113 Laurel Rd, Stratford, NJ, 08084, USA

**Keywords:** Polycystic ovary syndrome, Fractures, Bone, Bone density

## Abstract

**Background:**

Polycystic Ovarian Syndrome (PCOS) impacts reproductive-age women through metabolic disturbances and hormone imbalances. These changes have been linked to bone health, but the association between PCOS and incidence of fractures remains unclear. Clarifying the link between PCOS and fractures is important to guide clinical care and develop prevention strategies.

**Methods:**

A retrospective cohort analysis using the TriNetX research platform evaluated fracture incidence in female patients with and without PCOS. The study assessed the occurrence of distal radius, femoral neck, ankle, and metatarsal fractures, identified through ICD-10 codes. Fracture incidence was measured from one day following PCOS diagnosis to the most recent available encounter. Analysis was performed after propensity score matching, with statistical significance defined as a p-value of <0.05.

**Results:**

Final analysis included 174,937 patients in both cohorts. Incidence rates of distal radius, femoral neck, and metatarsal fractures were significantly reduced in patients with PCOS, with risk ratios of 0.7 (CI: 0.613-0.8), 0.647 (CI: 0.521-0.804), and 0.913 (CI: 0.834-0.998), respectively. Although the incidence rate of ankle fractures was also reduced in the PCOS cohort, this finding was not statistically significant (RR: 0.921, CI: 0.836-1.015).

**Conclusion:**

The results of this study show that patients with PCOS have lower distal radius, femoral neck, and metatarsal fracture rates compared to patients without PCOS. These findings suggest PCOS is not related to an increase in the risk of fractures. Further investigation is needed to better understand this potentially fracture-protective effect across different bone sites.

## 1 Introduction

Polycystic ovarian syndrome (PCOS) is a common endocrine disorder affecting women of reproductive age, characterized by hyperandrogenism, menstrual cycle irregularities, and infertility. In addition to its reproductive implications, PCOS is also known to cause metabolic changes, such as insulin resistance, obesity, chronic inflammation, and vitamin D deficiency, all which can have an impact on bone health.^1^^,^^2^ These effects persist into menopause, when women are most vulnerable to fragility fractures. Despite these well-established factors, the relationship between PCOS and incidence of bone fractures remains ambiguous and requires further investigation.

Current literature presents a complex and inconsistent understanding of the association between PCOS and fracture incidences, reflecting methodological variability and a lack of synthesis across studies. For example, some studies have reported a decrease in vitamin D levels in 67-85% of PCOS patients, potentially increasing susceptibility to low bone mineral density,^1^ while other hypotheses suggest that menstrual irregularities lead to low levels of estrogen and progesterone that may also negatively affect bone metabolism.^3^ In contrast, several studies have proposed that androgens and increased adiposity in women with PCOS may exert a protective role on bone density by promoting the conversion of androgens to estrogens.^4^, ^5^, ^6^

Despite prior contributions, critical gaps in knowledge remain, highlighting the need for large-scale, population-based studies to better understand fracture incidence in this population. To address this, the present study evaluates fracture incidence in women with and without PCOS using the TriNetX research platform, specifically the TriNetX U.S. Collaborative Network, an electronic health record database from healthcare organizations across the United States. The analysis focuses on distal radius, femoral neck, ankle, and metatarsal fractures, selected to include both classic fragility fractures and additional skeletal sites with differing bone composition and hormonal sensitivity, allowing for a comprehensive evaluation of potential site-specific effects of PCOS on fracture risk. It was hypothesized that women with PCOS would exhibit an increased risk of fractures compared with matched controls. Understanding these associations can provide novel insights into the impact of PCOS on bone health and inform strategies for long-term prevention care.

## 2 Methods

### 2.1 Study design

A retrospective cohort study was conducted using the TriNetX U.S. Collaborative Network, a database of de-identified electronic health records from participating healthcare organizations within the United States. This platform is Health Insurance Portability and Accountability Act (HIPAA) compliant and is Institutional Review Board exempt due to the absence of patient identifier information. TriNetX is a subscription-based network that is not publicly accessible, with access limited to affiliated institutions that have established agreements with the platform.

Two patient cohorts were created using International Classification of Diseases, 10th edition (ICD-10) diagnosis code. Both cohorts included female patients, defined by sex assigned at birth based on external anatomy, who had at least one documented encounter for a general adult medical examination (Z00.0). Patients were classified as PCOS + if they had at least one recorded ICD-10 diagnosis for PCOS (E28.2) at any time in their electronic health record. Patients were classified as PCOS- if no diagnosis code for PCOS (E28.2) was present in their medical record. Diagnosis codes were obtained from all available inpatient and outpatient encounters within the TriNetX network. Exclusion criteria included fractures that occurred more than 20 years prior to the index event.

The incidences of distal radius, femoral neck, ankle, and metatarsal fractures were compared between both cohorts using corresponding ICD-10 codes. Distal radius fractures were defined using codes S52.501A–S52.569B, including Colles', Smith's, Barton's, greenstick, torus fractures, as well as open and closed injuries across both wrists. Femoral neck and proximal femur fractures were identified using codes S72.001A–S72.146C to include fractures of the femoral head, neck, trochanteric, intertrochanteric, and subtrochanteric regions. Ankle fractures were defined using codes S82.51XA–S82.856C to include displaced and nondisplaced fractures of the medial, lateral, bimalleolar, and trimalleolar regions for both open and closed injuries. Metatarsal fractures were identified with codes S92.301A–S92.356B, representing displaced and nondisplaced fractures of the first through fifth metatarsals, including open and closed injuries across both feet. Fracture incidence was measured from one day following the PCOS diagnosis to the most recent available patient encounter.

Propensity score matching was performed within the TriNetX platform in a 1:1 ratio to balance baseline characteristics between PCOS and non-PCOS cohorts, thereby reducing potential bias from demographic and clinical differences. Matching included selected covariates, including age, race, body mass index (BMI), type 2 diabetes (T2DM), tobacco use, vitamin D deficiency, steroid use, and osteoporosis, all of which have been shown in prior studies to influence both bone health and PCOS outcomes. Covariate balance was evaluated using the platform's built-in assessment tools.

### 2.2 Data analysis

Statistical analysis was performed within the TriNetX platform. Cohorts were compared using measures of association, including risk ratios (RRs) and odds ratios (ORs), each with 95% confidence intervals. Fracture incidence rates were adjusted for potential confounders, including age, race, BMI (30-39, 40+), T2DM, tobacco use, vitamin D deficiency, long-term steroid use, and osteoporosis. Statistical significance was defined as a p-value of <0.05.

## 3 Results

The PCOS + cohort included 193,630 women and the PCOS- cohort included 7,768,325 women. Population characteristics prior to matching are summarized in Table 1. After exclusions and propensity matching, final analysis included 174,937 patients in each cohort.

**Table 1:** Population characteristics pre- and post-propensity matching.

Characteristic	PCOS	Post-Match PCOS	Non-PCOS	Post-Match Non-PCOS
**N (after exclusion)**^a^	176,261	174,937	7,140,958	174,937
**Age at Index (mean ± SD)**	32 ± 9.57	32 ± 9.57	45.7 ± 19.7	32 ± 9.62
**White**	119,304 (68%)	119,277	6,952,827 (68%)	119,215
**Black or African American**	22,929 (13%)	22,927	931,139 (13%)	23135
**Asian**	8813 (5%)	8813	332,883 (5%)	8707
**BMI ≥ 40**	25,688 (15%)	25,656	180,125 (3%)	25,769
**BMI 30**–**39**	24,052 (14%)	24,020	314,168 (5%)	24,323
**Type 2 Diabetes Mellitus**	17,726 (10%)	17,699	557,297 (8%)	17,714
**Tobacco Use**	4803 (3%)	4797	130,407 (2%)	4817
**Osteoporosis (no fracture)**	1034 (1%)	1034	287,524 (4%)	954
**Vitamin D Deficiency**	29,444 (17%)	29412	574,778 (8%)	29316
**Long-term Steroid Use**	3743 (2%)	3737	94,793 (1%)	3777

a Patients meeting the index event criteria more than 20 years ago were excluded.

The incidence of distal radius fractures was found to be significantly lower in the PCOS + cohort compared to the PCOS- cohort (0.21% vs 0.3%). Likewise, the incidence of femoral neck fractures were found to be significantly lower in the PCOS + cohort compared to the PCOS- cohort (0.077% vs 0.118%). The incidence of metatarsal fractures was found to be significantly lower in the PCOS + cohort compared to the PCOS- cohort (0.519% vs 0.569%). Though the incidence of ankle fractures was found to be lower in the PCOS + cohort, there was no statistical difference. These results are summarized in Table 2.

**Table 2:** Fracture Incidence and Risk Estimates of PCOS vs. Non-PCOS Cohorts.

Fracture location	PCOS	No PCOS	Risk Ratio (CI)	Odds Ratio (CI)	p-value
N	174,937	174,937			
**Distal radius**	367 (0.21%)	524 (0.3%)	0.7 (0.613–0.8)	0.7 (0.612–0.8)	<0.001
**Femoral neck**	134 (0.077%)	207 (0.118%)	0.647 (0.521– 0.804)	0.647 (0.521–0.804)	<0.001
**Ankle**	780 (0.446%)	847 (0.484%)	0.921 (0.836– 1.015)	0.921 (0.835–1.015)	0.0959
**Metatarsal**	908 (0.519%)	995 (0.569%)	0.913 (0.834– 0.998)	0.912 (0.833–0.998)	<0.05

## 4 Discussion

In this large retrospective cohort study, women with PCOS demonstrated lower rates of distal radius, femoral neck, and metatarsal fractures compared to matched cohorts, while ankle fractures occurred at the same incidence. Although several of these differences reached statistical significance, the absolute differences in fracture rates were small, suggesting the clinical impact at the individual patient level may be limited.

Prior studies exploring fracture rates in patients with PCOS have been inconsistent, likely due to differences in study methodology, heterogeneity of hormone profiles in PCOS patients, or the varying impact of PCOS on different skeletal sites. In line with this study's findings, a previously conducted large registry study has reported reduced risk of appendicular fractures in women with PCOS.^6^ In contrast, a more recent population-based study has reported an increased risk of forearm fractures in this population.^3^ Although this study observed fewer femoral neck fractures among PCOS patients, previous meta-analyses have also shown mixed results, with some reporting lower bone mineral density at the femur in female patients with PCOS.^7^^,^^8^ Notably, the effect of PCOS pathophysiologically diverges at low and high BMIs, as described by Rissetti et al. At BMIs below 27, bone density was decreased at vertebral and nonvertebral sites, while at BMIs above 27 the inverse was found.^8^ This reflects the protective effect of obesity on fragility fractures in the general population. Meanwhile, the relationship between PCOS and fracture risk at metatarsal and ankle sites remains poorly characterized by existing literature.

The potential protective relationship of PCOS on bone health observed in this study may be explained by the hormonal and metabolic characteristics of the condition. Studies have proposed that androgen excess in women with PCOS may exert direct effects on bone through activation of androgen receptors on osteoblasts, or indirect effects by enhancing peripheral aromatization of androgens to estrogens, particularly in the presence of increased adipose and stromal tissue.^9^^,^^10^ This conversion is significant, as estrogen is known to play an important role in maintaining bone mineral density.^11^^,^^12^ Similarly, studies in men with aromatase deficiency have shown that estrogen replacement can significantly increase bone mass.^9^ These findings support the concept that the combination of androgen excess and increased adiposity in PCOS patients may provide a protective mechanism against fractures by promoting higher estrogen levels and improving bone density.^7^^,^^8^

Although this study adjusted for T2DM, insulin resistance and hyperinsulinemia remain attributed to women with PCOS, even in the absence of a formal diabetes diagnosis, and may contribute to the observed protective effect against bone fractures. Insulin is known to have overall anabolic effects on bone through promoting osteoblast proliferation and collagen synthesis, which enhances bone formation and integrity.^11^ In women with PCOS specifically, past literature has found bone mineral density at the lumbar spine and femoral neck to be positively associated with greater insulin resistance levels.^12^ In this study, the incidence of femoral neck fractures was significantly lowered in the PCOS cohort, which may reflect the influence of these various factors. A combination of androgen excess, estrogen conversion, and insulin resistance likely plays a synergistic role to promote bone health.

Further contextualizing this study's results, past literature has demonstrated that volumetric bone density and structural parameters at different skeletal sites in women are differentially influenced by sex steroid levels.^13^ Particularly, trabecular-rich sites, such as the spine and femoral neck, have been found to be more sensitive to estrogen and androgen status than cortical-rich sites, such as the radius or metatarsals. This may explain why women with PCOS, a demographic often presenting with altered androgen and estrogen levels, show variable bone mineral density and fracture risk at different skeletal sites.^2^^,^^4^^,^^7^^,^^8^^,^^14^ The relative lack of data on metatarsal and ankle fractures in PCOS patients may reflect the lower sensitivity of these sites to alterations in sex steroid levels.

In postmenopausal women, current evidence shows that PCOS-related hyperandrogenism, along with other metabolic complications, can persist beyond menopause, yet no significant differences in bone mineral density or fracture incidence compared with age-matched controls have been noted.^15^, ^16^, ^17^ Direct studies specifically examining fracture risk in postmenopausal women with PCOS remain limited, likely because diagnosing PCOS at or after menopause can be difficult, which complicates study recruitment.^18^ Thus, although hyperandrogenism can persist in postmenopausal women with PCOS, the long-term impact of PCOS on bone health and fracture risk in this population remains uncertain and warrants further investigation.

This study's retrospective design and usage of the TriNetX database limit causal inference and introduce the potential for residual confounding despite propensity score matching. Important confounders, including physical activity, fall history, hormonal therapy, and menopausal status, were not available within the database and therefore could not be incorporated into the matching process. Additionally, lifestyle factors and medication history (e.g., hormonal contraceptives, bisphosphonates) may be incompletely captured due to reliance on provider coding.

The reliance on ICD-10 codes, which vary in usage across institutions and individual providers, may also result in misclassification of PCOS or fracture events. In particular, PCOS identification relied on ICD-10 coding (E28.2) rather than established clinical diagnosis criteria (e.g., the Rotterdam criteria), which may contribute to misclassification. Furthermore, the temporal relationship between PCOS diagnosis and fracture incidence was constrained by the database structure, as only fractures occurring after the index event of PCOS diagnosis were captured, potentially excluding those with undiagnosed PCOS. The duration of follow-up was not standardized or directly measured as person-time at risk, which may limit interpretation of fracture incidence and risk estimates. Outcomes were also not stratified by PCOS phenotype, severity, or menopausal status, factors that affect relevant hormonal and metabolic profiles. The absence of laboratory data (e.g., androgen, estrogen, insulin sensitivity, inflammatory markers, bone mineral density) further limits interpretation. Future studies should incorporate these variables to better characterize fracture risk and the long-term skeletal effects of PCOS.

Finally, while the observed differences in fracture risk reached statistical significance, absolute differences were small and may not be clinically meaningful. The large sample size inherent to TriNetX analyses can detect minor differences that are unlikely to translate into practical or patient-relevant outcomes. Therefore, the findings should be interpreted as hypothesis-generating rather than evidence of a clinically important association.

## 5 Conclusion

This retrospective, propensity-matched cohort study utilizing the TriNetX database demonstrated that women with PCOS had a significantly lower incidence of distal radius, femoral neck, and metatarsal fractures. The protective effect of PCOS on bone health may be attributed to factors such as androgen excess, increased adiposity, and insulin resistance. Further investigation is required to better understand the site-specific differences in bone fracture risk and to clarify how metabolic and hormonal factors uniquely play a role in bone health in women with PCOS.

## Credit author statement

**TL:** Conceptualization, Methodology, Formal Analysis, Data Curation, Writing - Original Draft, Writing - Review & Editing, Project Administration.

**WP:** Conceptualization, Writing - Original Draft.

**AS:** Methodology, Formal Analysis, Data Curation.

**SS:** Conceptualization.

**KD:** Writing - Review & Editing.

**AI:** Writing - Review & Editing, Supervision.

**MB:** Writing - Review & Editing, Supervision.

## Ethical approval statement

Not required due to use of the HIPAA-compliant, de-identified TriNetX U.S. Collaborative Network database, which is IRB-exempt.

## Guardian/patient’s consent statement

Not required due to use of the HIPAA-compliant, de-identified TriNetX U.S. Collaborative Network database, which is IRB-exempt.

## Funding

This research did not receive any specific grant from funding agencies in the public, commercial or not-for-profit sectors.

## Declaration of competing interest

The authors declare that they have no known competing financial interests or personal relationships that could have appeared to influence the work reported in this paper.
